# Recent advances in understanding RNA polymerase II structure and function

**DOI:** 10.12703/b/9-11

**Published:** 2020-11-17

**Authors:** Daniel Reines

**Affiliations:** 1 Department of Biochemistry, Emory University School of Medicine, Atlanta, GA, USA

**Keywords:** RNA polymerase II, condensate, low complexity, chromatin, bursting, elongation, termination

## Abstract

More than 50 years after the identification of RNA polymerase II, the enzyme responsible for the transcription of most eukaryotic genes, studies have continued to reveal fresh aspects of its structure and regulation. New technologies, coupled with years of development of a vast catalog of RNA polymerase II accessory proteins and activities, have led to new revelations about the transcription process. The maturation of cryo-electron microscopy as a tool for unraveling the detailed structure of large molecular machines has provided numerous structures of the enzyme and its accessory factors. Advances in biophysical methods have enabled the observation of a single polymerase’s behavior, distinct from work on aggregate population averages. Other recent work has revealed new properties and activities of the general initiation factors that RNA polymerase II employs to accurately initiate transcription, as well as chromatin proteins that control RNA polymerase II’s firing frequency, and elongation factors that facilitate the enzyme’s departure from the promoter and which control sequential steps and obstacles that must be navigated by elongating RNA polymerase II. There has also been a growing appreciation of the physical properties conferred upon many of these proteins by regions of each polypeptide that are of low primary sequence complexity and that are often intrinsically disordered. This peculiar feature of a surprisingly large number of proteins enables a disordered region of the protein to morph into a stable structure and creates an opportunity for pathway participants to dynamically partition into subcompartments of the nucleus. These subcompartments host designated portions of the chemical reactions that lead to mRNA synthesis. This article highlights a selection of recent findings that reveal some of the resolved workings of RNA polymerase II and its ensemble of supporting factors.

## Introduction

RNA polymerase II (Pol II) is an essential, multi-subunit, DNA-dependent, nucleotidyltransferase. In eukaryotes, Pol II is the one of three nuclear RNA polymerases. It is responsible for unspooling the genetic program in the form of protein-coding mRNAs and some small non-coding RNAs. Pol II’s activity is highly regulated at a number of steps, many of which impact the process of getting Pol II to transcription initiation sites. Pol II’s ability to disengage from the promoter and become committed to elongating nascent RNA is another complex regulated process, as is Pol II’s ability to complete that primary transcript. Finally, transcription termination, which involves Pol II choosing where to stop polymerization and releasing its RNA product, can be modulated. Understanding the regulation and intricacies of all three stages of the transcription cycle—initiation, elongation, and termination—has been the focus of studies for decades, yet new aspects of these processes continue to be revealed. Several of the newest developments are described here.

## Cryo-electron microscopy of RNA polymerase II assemblages: a close look at large objects

A major advance at the turn of the 21st century was the development of atomic-level structural maps of the approximately 0.5 megadalton Pol II enzyme by using x-ray crystallography^1–3^. Cryo-electron microscopy (cryo-EM) refined and expanded these initial structural models, as well as those of the comparably large TFIIH and TFIID proteins, which are the most complex of the general initiation factors that Pol II uses to locate promoters and initiate transcription^4,5^. Even larger assemblies of Pol II in which it is bound to some of its general initiation factors have also been studied using cryo-EM^6–16^. More recently, cryo-EM has been exploited to study more elaborate transcription complexes, including Pol II associated with its chromatin template, its RNA product, and some attendant proteins that guide it through the transcription cycle. Reports that provided mechanistic insight into these functionally important assemblies will be described in this section.

### The RNA polymerase II–nucleosome confrontation

An age-old question in the transcription field has been: what happens when template-engaged Pol II encounters a nucleosome? Nucleosome-wrapped duplex DNA is an impediment to Pol II, particularly given the enzyme’s need to transiently separate the DNA strands. Certain elongation factors, however, can facilitate nucleosomal readthrough. Kujirai *et al*. resolved seven intermediates of Pol II in the act of encountering, and progressively moving through, a nucleosome with the aid of elongation factor TFIIS^17^. This elongation factor activates a nascent transcript nuclease activity in Pol II that aids in its passage through obstacles to elongation^18,19^. Cryo-EM analysis showed that Pol II stalls at the entrance to the nucleosome but that the nucleosome remains structurally unchanged. Further translocation allows it to penetrate the DNA–histone interaction in a fashion that peels DNA off the histone octamer. Complete passage through the nucleosome was not monitored, but extrapolation from these structures suggests that Pol II could proceed into a form in which nucleosomal DNA is looped over polymerase or in which the nucleosome has lost a subset of histones; both are intermediates previously proposed to exist during Pol II transit^20^. The addition of the general elongation factors Elf1 and Spt4/5 (also known as DRB sensitivity-inducing factor or DSIF) synergistically lowered the barrier of Pol II entry into the nucleosome by interposing themselves between the enzyme and the nucleosome^21^. Pol II advanced further as the nucleosome disk became tilted while the collection of bound elongation factors acted as a chisel to displace DNA from the nucleosome. This effectively averts Pol II from getting trapped between the DNA and histones. As more structures of Pol II–elongation factor complexes are solved, it is likely we will learn that different elongation factors and different combinations of factors help Pol II surmount the nucleosome obstacle by distinct mechanisms.

### RNA Polymerase II and a dynamically changing set of elongation factors

Pol II assembles into a large pre-initiation complex where it engages a region of nucleosome-free DNA abetted by a collection of general initiation factors. RNA chain polymerization is accompanied by Pol II’s disengagement from this tight complex. This is quickly followed by Pol II’s association with elongation factors that stabilize the just-initiated Pol II, thereby holding it in a paused condition analogous to an idling automobile. The decision to abort elongation, versus complete the primary transcript, is a determinant of transcriptional output and a regulated event. This process may be necessary for the nascent RNA capping machinery to engage Pol II while awaiting the maturation of the complex mediated by a specific set of kinases. Two factors key to generating the paused state are the multi-subunit proteins, negative elongation factor (NELF) and DSIF, whose biochemical activities as Pol II elongation modulators were identified many years ago^22–24^. Cryo-EM analysis now reveals structural details of how these two proteins engage elongating Pol II^25^. Importantly, the positions of the docked elongation factors appear to be mutually exclusive with the initiation factors that recruit Pol II to DNA, delineating a structural variance that differentiates the pre-initiated and initiated states. Prior work showed that DSIF provides a DNA clamp and an RNA clamp that preserve the transcription bubble and guide the RNA through its exit tunnel on Pol II^26,27^. The consequence of NELF addition is to render the complex elongation-incompetent, possibly because the complex gains an anomalous positioning of the DNA:RNA hybrid substrate that is unsuitable for templating or allowing access and retention of nucleoside triphosphate substrates in the active site^25^. These proteins may also occlude the binding of other positively acting elongation factors. Recent findings enabled by a NELF-depletion technique in cultured human cells indicated that NELF’s restriction of RNA extension by Pol II is not the only yoke put on polymerase but that an additional unidentified constraint is apparent before the fully elongation-competent enzyme is liberated^28^. Since NELF-associated pol II is an intermediate to productive elongation, the absence of NELF may also have precluded any necessary downstream positive events from taking place.

The cryo-EM analysis of transcribing Pol II was extended by assembling elongation complexes in the presence of a variety of well-studied, biochemically characterized proteins involved in elongation^29^. These include positive elongation factor b (P-TEFb), a cyclin-kinase^22^ that phosphorylates and releases NELF from the complex^30,31^, and the RNA polymerase-associated factor (Paf1) complex^32,33^, whose mode of action in stimulating elongation is complicated and unresolved. Also, the association of Spt6, one of a number of Spt transcription factors that were revealed in a landmark productive and penetrating genetic suppressor screen (recall that DISF is composed of Spt4 and Spt5), was studied^34^. An activated elongation complex containing DSIF, PAF, and Spt6 was assembled with P-TEFb and ATP^29^. The action of the P-TEFb kinase facilitates the replacement of pause-stabilizing NELF with the PAF complex, thereby removing NELF’s ability to misposition the nascent RNA:DNA hybrid and limit substrate NTP accessibility, as described above. Stimulation by PAF and Spt6 could be due to these proteins’ coating of the surface of Pol II and an allosteric conformational change that may facilitate elongation by assisting template annealing behind the enzyme. Spt6 engages with the activated elongation complex near the RNA exit site following P-TEFb’s specific phosphorylation of Pol II. PAF and Spt6 appear to remodel the extent of DSIF’s clamping of DNA and RNA at their respective exit channels. This kinase is profligate indeed, as it can phosphorylate all the components described in these Pol II–“plus” structures: DSIF, NELF, PAF, Spt6, and polymerase itself. This places the kinase at the hub of coordinating the elongation complex’s gain and loss of proteins at an important regulated step of transcription possessed by many genes, namely, promoter escape. A recent cryo-EM study of the activated complex with trapped Rtf1, an otherwise dissociable subunit of the PAF complex, suggested that Rtf1 provokes an additional conformational change that may enhance Pol II translocation, thereby facilitating elongation^35^.

## Bursting: intermittent RNA polymerase II firing from promoters

Over the years, most investigations studying Pol II have examined readouts from populations of polymerase molecules and cells. The advent of high-resolution microscopy and biophysical techniques has allowed the behavior of individual molecules or templates to be observed. These advances have been particularly valuable for populations of nucleic acid polymerases because individual molecules can asynchronously occupy distinct phases of their polymerization cycle. In fact, some molecules may be altogether inactive at any point in time. Thus, a surprising heterogeneity in performance can be observed in otherwise identical molecules; that is, the various polymerases or templates in a population can carry out different functions and unless each can be resolved, a population average is obtained. One such heterogeneity is transcriptional bursting. This is seen when a single promoter releases a volley of polymerase initiations which alternates with relatively quiescent intervals^36,37^. The rate of firing during a burst, or the length of the interval between bursts, is variable and subject to regulation.

The development of single-molecule techniques has led to advances in understanding the molecular basis for bursting and its regulation. Recently, Bartman *et al*. used both single-cell and ensemble methods to show that biological stimuli accelerate Pol II pause release and bursting and, somewhat counterintuitively, not Pol II recruitment rates^38^. In other words, Pol II is driven to a promoter during bursting in contrast to the idea that bursting results from pre-loaded polymerases.

Using computational modeling and single-cell RNA sequencing, investigators were able to dissect burst size and frequency underlying genome-wide transcription^39^. This analysis suggested that enhancers control burst frequency, consistent with early models that enhancers increase the probability of Pol II firing^40,41^. Promoters appeared to govern burst size, and TATA-containing sequences directed larger bursts than promoters lacking the TATA consensus, again as suggested some years ago^42,43^. These findings imply that genotypic and cell type differences can yield alternative burst sizes, and hence gene output, as a function of the selection of proteins that engage a locus as a consequence of its DNA sequence.

Bursting has also been explored by using sophisticated computational and optical nanoscopy techniques aided by target-locking and background suppression methods^44^. This approach enabled the study of transcription of single genes in individual living cells with the ability to follow Pol II and its transcription factors in compartments of extremely small volumes. For the *Nanog* gene, bursting was accompanied by clustering of Pol II with the Sox2 and Brd4 proteins at the locus in a manner consistent with looping of the enhancer and promoter thereby forming a bridge between enhancer-bound proteins in contact with promoter-bound Pol II. The size of the Brd4 cluster correlated with burst frequency similar to the relationship described above for enhancer-powered bursting. Further development of this technology promises the possibility of watching in real time a single Pol II molecule, and its support factors, advance through the entire transcription cycle.

## Condensates: compartmentalization of the transcription machinery

An active area of research has been the examination of how subcellular compartments form from self-assembling proteins that contain low-complexity domains (LCDs) (that is, stretches of amino acid sequence of biased composition)^45^. A well-studied paradigm is the case of cytoplasmic stress granules, a non-membrane-delimited compartment that is a site of RNA sequestration and metabolism. The LCDs of RNA-binding proteins support liquid–liquid phase separation and contribute to the assembly of these compartments.

This concept has been extended to the machinery that activates and carries out transcription, including Pol II and the Mediator complex, as well as proteins that co-transcriptionally modify and terminate the primary transcript (reviewed in 46–48). A model has emerged in which the low-complexity heptapeptide repeat domain of Pol II found on the C-terminus of its largest subunit, enables the enzyme to enter and exit condensates as a function of its phosphorylation state. The condensates represent a chromatin-associated, changing set of transcript-modifying and -processing enzymes that handle the nascent RNA and often possess their own LCDs. This improved description of foci in which the steps of mRNA biogenesis take place in a concerted manner has been aided by technical advances, including live cell imaging, and refines earlier ideas of transcription “factories” (reviewed in 49).

Two notable extensions of the concept were reported in the last year in findings that emphasized how condensate formation operates across the transcription cycle. Gallego *et al*.^50^ showed that ubiquitination of histone H2B lysine 123, a modification associated with active chromatin, is stimulated through biomolecular condensation mediated by the LCD of a specific ubiquitination complex-associated protein. Through the organizing principle of phase separation, the ubiquitination apparatus becomes co-localized with nucleosomal H2B in what the authors refer to as a “reaction chamber”^50^. This process operates broadly across the nucleosomes of the body of genes in a process that is poorly understood.

Meanwhile, Guo *et al.*^51^ provided evidence from mammalian cells that Pol II with a hypo-phosphorylated CTD joins Mediator condensates established through enhancer sequences. Once the CTD becomes phosphorylated, Pol II is dislodged from that condensate and elongating Pol II forms a condensate with splicing factors in a spatially separate locale. The overall importance of these condensation reactions requires further study. Future work will be needed to validate what seems to be a recurring theme of a handoff of Pol II between a chain of condensates. By linking condensates, various parts of the mRNA biogenesis pathway could be connected by what is effectively a substrate channeling mechanism.

## RNA Polymerase II brakes into the termination zone

There has been continued progress in dissecting the polyadenylation-coupled termination process carried out by Pol II. A prevailing idea for how Pol II terminates transcription at the end of protein-encoding genes is the so-called “torpedo” model^52,53^. In this durable, three-decade-old proposal, the precursor transcript is endonucleolytically cut just after Pol II transcribes the polyadenylation signal into nascent RNA^54^. The 3′ terminus of the upstream piece is polyadenylated and becomes the mature mRNA. Importantly, Pol II continues extending the downstream fragment until an exoribonuclease engages the still-emerging transcript and begins hydrolyzing it in the 5′-to-3′ direction while advancing toward Pol II, effectively using the RNA as a “trail of breadcrumbs” to home in on and chase down the still-translocating polymerase. The nuclease acts as a torpedo by contacting, and ultimately displacing, Pol II from the template, thereby terminating transcription. A key feature of the model is the kinetic competition in which the digesting nuclease “catches” elongating polymerase.

We now learn^55^ that phospho-Spt5, which piggybacks on Pol II starting at DSIF’s engagement with the paused polymerase^56^, is de-phosphorylated just after Pol II transcribes the polyadenylation site^55,57,58^. This switches Pol II, which accelerated out of the promoter region because of Spt5’s phosphorylation^56,59,60^, into a slower elongating form which facilitates the nuclease’s ability to overtake Pol II and trigger termination^55^. The exact mechanism by which de-phosphorylation of Spt5 slows Pol II remains to be elucidated. An interesting related finding is that the initial loading of Spt5 onto Pol II at the promoter was found to employ the c-Myc proto-oncoprotein^60^, which has been long studied as a DNA-binding transcription factor but is also known to play a general role in elongation^61–64^. c-Myc’s effect on normal and pathological changes in gene expression could be operating at least in part through a widespread boosting of the output of active genes through Spt5’s elongation-stimulating activity.

## Elongation and termination by RNA polymerase II help set chromatin organization

Aided by powerful molecular techniques, we have learned much about the physical basis for the functional arrangement of chromatin during the last decade. Distant portions of the chromosome that contact each other segregate chromatin into functionally distinct topologically associating domains^65–68^. The resulting loops use specific proteins to section chromatin into transcriptionally active and inactive segments^69^. In 2018, Heinz *et al*. showed how transcription can remodel the boundaries of these looped domains^70^. By exploiting a viral protein that broadly disrupts host transcription termination, the authors found that readthrough by Pol II of the end of the transcription unit resets loop boundaries, seemingly plowing the proteins off chromatin and making active regions out of formerly inactive ones. In contrast, inhibition of elongation led to re-association of distant sequences and compaction, thereby switching a formerly active region to an inactive one. Thus, while chromatin is generally remodeled to become permissive for transcription, transcription can also remodel the three-dimensional organization of chromatin.

## Summary and outlook

A half century after RNA Pol II was first extracted and purified from eukaryotic cells^71^, we are still learning about the enzyme, its auxiliary factors, and the environment in which it operates. A large international research effort armed with a sophisticated toolbox of experimental methods has revealed many of the detailed mechanistic steps that Pol II employs to find, transcribe, and disengage from eukaryotic genes. Many of the participating protein and nucleic acid components have been identified, and important regulatory post-translational modifications have been characterized. Biophysical techniques enabling the study of single Pol II molecules, and cryo-EM which has provided relatively high-resolution structures of very large complexes, have propelled this decade’s progress. These approaches and their future refinement should continue to be fruitful in placing transcribing Pol II in its three-dimensional nuclear location and in characterizing the dynamics of what proteins and nucleic acids enter and exit the transcription domain and how the act of transcription remodels the chromatin/nuclear environment. A goal will be to describe the numerous dynamic steps of the transcription cycle, including the co-transcriptional events that engage the elongation complex, with the objective of filling out our picture of this fundamental pathway in its cellular context.
